# The effectiveness of Du moxibustion for chronic obstructive pulmonary disease: A protocol for systematic review and meta-analysis of randomized clinical trials

**DOI:** 10.1097/MD.0000000000024935

**Published:** 2021-03-19

**Authors:** Yue Ruizhen, A. Rigun, Li Fu, Chen Baoshan, Xie Dingyi, Xiong Jun, Huang Xianbao, Rixin Chen

**Affiliations:** a Jiangxi University of Traditional Chinese Medicine; b Affiliated Hospital of Jiangxi University of Traditional Chinese Medicine, Nanchang, China.

**Keywords:** chronic obstructive pulmonary disease, Du moxibustion, protocol, systematic review

## Abstract

**Background:**

As a common respiratory disease, Chronic Obstructive Pulmonary Disease (COPD) develops progressively. Du moxibustion can effectively treat COPD, and no adverse reactions have been reported. This research mainly evaluated the efficacy and safety of Du moxibustion in the treatment of COPD.

**Methods:**

Seven databases (PubMed, Cochrane Library, Embase, China National Knowledge Infrastructure (CNKI), Chinese Biomedical Literatures Database (CBM), Wanfang Database (WF), Chinese Scientific Journal Database (VIP)) will be searched for all relevant eligible randomized controlled trials (RCTs) from the date of establishment to January 6, 2021. No matter whether they were blind or not, and regardless of the language and type of publication, these experiments could be included. Two authors (YRZ, ARG) will search the database respectively, extract relevant data, and use the Cochrane bias risk tool to evaluate the quality of the literature. RevMan V5.3 software will be used for data processing.

**Results:**

The results of this research are mainly used to evaluate the efficacy and safety of Du moxibustion in the treatment of COPD.

**Conclusions:**

This systematical review is expected to provide evidence-based and valuable suggestions for Du moxibustion in the treatment of COPD.

**Study registration number:**

INPLASY202110045.

## 1 Introduction

Chronic obstructive pulmonary disease (COPD) is a chronic progressive respiratory disease characterized by irreversible airflow limitation, which can lead to chronic respiratory failure in severe cases.^[1]^ Approximately 300 million people in the world are suffering from COPD, most in developing countries.^[2]^ At present, COPD ranks the fourth among the diseases that cause global population death, and it is likely to leap to the third.^[3]^ The main cause of COPD is inhalation of poisonous gas and genetic factors.^[1]^ In developed nations, smoking is the main cause. In developing nations, cooking and occupational exposure are the main risk factors.^[4]^ The main symptoms of COPD include chronic cough, excessive mucus secretion, and dyspnea.^[5]^ Now we mainly use inhaled bronchodilators, anticholinergic drugs, and corticosteroids to treat COPD. But this kind of treatment requires long-term drug use, and the later drug resistance results in the increase of the dose increase the dose, and the side effects of this treatment are also very clear.^[6]^

Moxibustion, as a momentous component of Traditional Chinese Medicine (TCM), belongs to the scope of external treatment of traditional Chinese medicine. Moxibustion can warm the channels and dissipate cold, activate blood circulation and dredge collaterals, promote blood circulation, relieve cough and relieve asthma.^[7]^ As a kind of moxibustion, Du moxibustion mainly acts on the first lateral line of Du Meridian and foot Taiyang bladder meridian.^[8]^ The main characteristics of moxibustion are wide area and strong firepower.^[9]^ In addition, Du moxibustion can regulate the Qi and blood of meridians, assist and replenish Yang, enhance the body's healthy and plays to resist evil, and make Yin and Yang to be harmonious with each other, so as to achieve the goal of treating diseases.^[10]^

As a special moxibustion method, Du moxibustion is widely used in respiratory diseases, which can relieve the symptoms of cough, expectoration and dyspnea in patients with COPD.^[11]^ However, there is no systematic meta-analysis to prove the effectiveness and safety of Du moxibustion in the treatment of COPD. Therefore, in this paper an evidence-based medicine plan will be made for the clinical efficacy and safety of Du moxibustion in the treatment of COPD so as to provide evidence-based basis for the treatment of COPD in the future.

## 2 Methods

### 2.1 Inclusion criteria for study selection

#### 2.1.1 Types of studies

All clinical RCTs of Du moxibustion in the treatment of COPD will be selected, no matter in Chinese or English, no matter in what kind of journals.

#### 2.1.2 Types of participants

COPD patients included in this research need to be diagnosed first. No matter what type of COPD patients were diagnosed, they could be included regardless of gender, age, nationality, or education background. Those people allergic to moxibustion, intolerant of moxa smoke, or those with ulcers at the moxibustion site should be excluded.

#### 2.1.3 Types of interventions

The observation group can be treated with single Du moxibustion or combined with other therapies, such as pharmaceuticals, Chinese Herbal Medicine, needling, etc. The treatment of the control group can use simple western medicine, Chinese medicine, placebo, other therapies, or no treatment. The duration of Du moxibustion, the course of treatment and the intervals or frequencies of follow-up are not limited.

#### 2.1.4 Types of result measurement

##### 2.1.4.1 Primary results

The main results included clinical efficacy, clinical symptoms of COPD, and changes of pulmonary function indexes.

##### 2.1.4.2 Other results

Other results include the following:

1. Six minute walk test (6MWT).
2. Blood gas analysis indexes: PaO_2_, PaCO_2_, SaO_2_.
3. The levels of serum CRP, IL-8, and T cells (CD4+, CD8+) were measured.

### 2.2 Search methods for the identification of studies

Seven databases (PubMed, Cochrane Library, Embase, China National Knowledge Infrastructure (CNKI), Chinese Biomedical Literatures Database (CBM), Wanfang Database (WF), Chinese Scientific Journal Database (VIP)) will be searched for all relevant eligible randomized controlled trials (RCTs) from the date of establishment to January 6, 2021.

Search terminologies include diseases (Pulmonary Disease, Chronic Obstructive, chronic obstructive lung disease, chronic obstructive pulmonary diseases, COPD, chronic obstructive pulmonary disease) and intervention (Du moxibustion, dragon moxibustion, long snake moxibustion), and research types (randomized controlled trial OR randomized OR controlled clinical trial). Both MESH words and free words need to be searched. The search strategy of PubMed is shown in Table 1.

**Table 1:** Search strategy used in PubMed database.

Order	Strategy
#1	Search: “Pulmonary Disease, Chronic Obstructive”[Mesh] Sort by: Most Recent
#2	Search: Pulmonary Disease, Chronic Obstructive [Title/Abstract]
#3	Search: Chronic Obstructive Lung Disease [Title/Abstract]
#4	Search: Chronic Obstructive Pulmonary Diseases [Title/Abstract]
#5	Search: COPD [Title/Abstract]
#6	Search: Chronic Obstructive Pulmonary Disease [Title/Abstract]
#7	Search: #1 OR #2 OR #3 OR #4 OR #5 OR #6
#8	Search: Du moxibustion [Title/Abstract]
#9	Search: long snake moxibustion [Title/Abstract]
#10	Search: dragon moxibustion [Title/Abstract]
#11	Search: #8 OR #9 OR #10
#12	Search: “Randomized Controlled Trial” [Publication Type] Sort by: Most Recent
#13	Search: “Controlled Clinical Trial” [Publication Type] Sort by: Most Recent
#14	Search: random trials [Title/Abstract]
#15	Search: placebo [Title/Abstract]
#16	Search: #12 OR #13 OR #14 OR #15
#17	Search: #7 AND #11 AND #16

In addition, WHO international registry center of clinical trials and the Chinese clinical trial registry center also need to be searched to ensure that the literature can be as comprehensive as possible.

### 2.3 Data collection and analysis

#### 2.3.1 Selection of researches

**Figure 1:**
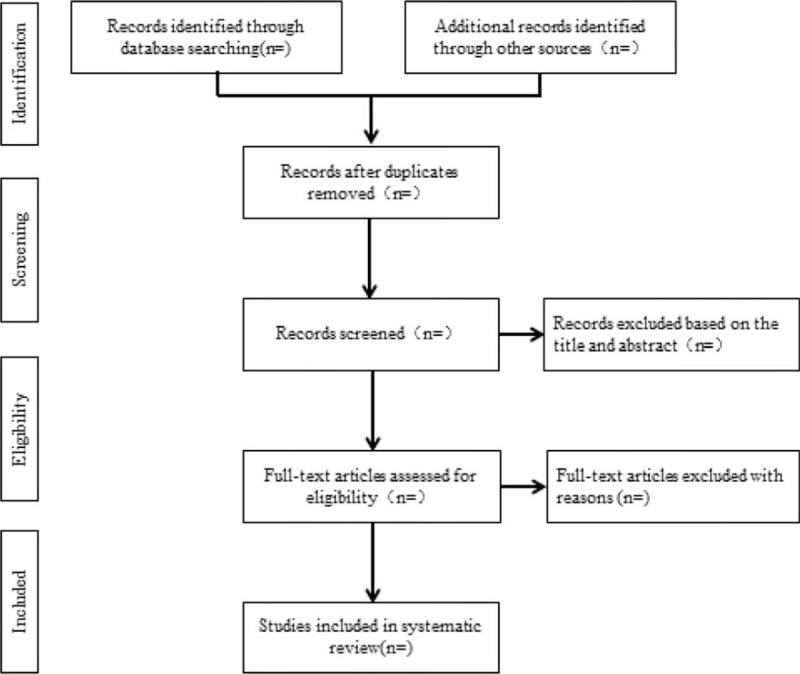
Flow diagram of study selection process.

We will import the searched literature transcripts in batch into the Note express 3.2.0 software and remove the duplicates. Then, topics, abstracts, full texts of the literatures will be reviewed by 2 authors independently, those eligible literatures will be selected according to the selective standard and eliminated criteria, and in case of disagreement, the third author will be arranged for adjudication. The process of this study is illustrated in Figure 1.

#### 2.3.2 Data extraction

Two assessors will design a table to extract data from those eligible RCTs. The contents of the table mainly include the first author, publication date, sample size and age mean of the sample, whether the blinding method was used, grouping method, interventions, treatment methods, treatment courses, follow-up time, treatment outcomes, and adverse reactions. Extraction of data will be done by 2 reviewers individually. If there is any objection, the third reviewer (HXB) will participate in the negotiation. If essential data are deficiency in the literature, the corresponding author will be contacted for information.

### 2.4 Risk of bias assessment

We will apply the Cochrane risk of bias tool to evaluate the quality of the included researches. It mainly includes the following items: whether the grouping scheme is really random, the allocation scheme is hidden, the blind method is used in the research process, the inclusion criteria are detailed, the baseline between groups is comparable, the outcome data is complete, and other possible bias risks. According to the evaluation, the results can be divided into 3 categories: risk of high bias, risk of low bias, or risk of unclear bias.^[12]^ When there exist differences, we will ask a third judge (CRX) to make a ruling.

### 2.5 Quantitative data synthesis and statistical methods

#### 2.5.1 Data analysis

RevMan V 5.3 software will be used for data analysis and forest map will be made. If the data is a binary variable, the odds ratio will be used, if the data is a continuous variable, the standardized mean difference will be used. We will select fixed or random effect models, according to the heterogeneity test results of this research.

#### 2.5.2 Heterogeneity test

The purpose of heterogeneity test is to check whether the results of each of the independent research is combinable.^[13]^ Q and *I*^*2*^ statistics can be used to detect both the existence and the degree of heterogeneity. If *I*^*2*^ < 50% and *P* > .1, we will ignore the heterogeneity between them and take the data as homogeneous, if *I*^*2*^ ≥ 50% or *P* < .1, the data is highly heterogeneous. At this moment, we need to explain the heterogeneity, and use subgroup analysis, meta-regression, or sensitivity analysis to find out the reasons for the heterogeneity.

#### 2.5.3 Subgroup analysis

Once we find high heterogeneity in the above heterogeneity test, we will analyze the data according to the TCM syndrome differentiation of COPD and the intervention methods of the control group.

#### 2.5.4 Meta-regression

When the heterogeneity is significant, we need to carry out Meta-regression to explore the source of heterogeneity. Meta-regression model was established to screen out the influencing factors of heterogeneity. Subgroup analysis based on this factor can significantly reduce the heterogeneity.

#### 2.5.5 Sensitivity analysis

If the heterogeneity is high, we need to exclude low-quality, small sample studies and then conduct a meta-analysis again to compare with the results of the non-exclusion meta-analysis. On if the result does not substantially change any more, can it be viewed as credible. On the contrary, we should be very careful in explaining the results and drawing conclusions.

#### 2.5.6 Publication bias

Funnel plot will be used to judge whether there exists publication bias. If the graph is a symmetrical inverted funnel, it means there is no publication bias; otherwise, the publication bias does exist.

#### 2.5.7 Grading the quality of evidence

The quality of evidence for each research will be appraised by 2 reviewers using a scoring tool independently.^[14]^ According to the consistency, limitation, accuracy, indirectness and publication bias of the selected literatures, the qualities will be graded, and finally divided into 4 grades: extremely low, low, moderate, and high.^[15]^

## 3 Discussion

As a kind of moxibustion, Du moxibustion also belongs to external treatment of traditional Chinese medicine, which is widely used in clinic. Studies have found that there are more than 40 diseases suitable for Du moxibustion treatment. As one of the more common respiratory diseases, moxibustion is also of advantages in treating COPD.^[16]^ At present, the RCTs of Du moxibustion in the treatment of COPD have confirmed that Du moxibustion has a significant effect in the treatment of COPD.^[17,18]^ However, there is no systematic review of Du moxibustion in the treatment of COPD. In this research, systematical evaluation is made mainly on the availability and safety of Du moxibustion in the treatment of COPD, in order to provide evidence-based basis for Du moxibustion in the treatment of COPD. However, there may be potential limitations to this study. For example, the sample size of the included trials, the strictness of the RCTs, the blind method, the severity of COPD, the time of single treatment, the course of treatment, and the different operation methods may exert influence on the results of the systematic review simultaneously.

## Author contributions

**Data curation:** Ruizhen Yue, Rigun A.

**Formal analysis:** Ruizhen Yue, Rigun A.

**Investigation:** Rigun A, Baoshan Chen.

**Methodology:** Rigun A, Fu Li.

**Project administration:** Ruizhen Yue, Xianbao Huang.

**Software:** Rigun A, Fu Li.

**Supervision:** Xianbao Huang, Rixin Chen.

**Validation:** Xianbao Huang, Fu Li.

**Visualization:** Baoshan Chen, Fu Li.

**Writing – original draft:** Ruizhen Yue, Dingyi Xie.

**Writing – review & editing:** Ruizhen Yue, Jun Xiong.
